# An Integrated Taxonomic Approach Points towards a Single-Species Hypothesis for *Santolina* (Asteraceae) in Corsica and Sardinia

**DOI:** 10.3390/biology11030356

**Published:** 2022-02-23

**Authors:** Paola De Giorgi, Antonio Giacò, Giovanni Astuti, Luigi Minuto, Lucia Varaldo, Daniele De Luca, Alessandro De Rosa, Gianluigi Bacchetta, Marco Sarigu, Lorenzo Peruzzi

**Affiliations:** 1 Department of Biology, University of Pisa, 56126 Pisa, Italy; paola.degiorgi@phd.unipi.it (P.D.G.); lorenzo.peruzzi@unipi.it (L.P.); 2 Botanic Garden and Museum, University of Pisa, 56126 Pisa, Italy; giovanni.astuti@unipi.it; 3 Environment and Life Sciences (DISTAV), University of Genoa, 16132 Genoa, Italy; luigi.minuto@unige.it (L.M.); lucia.varaldo@edu.unige.it (L.V.); 4 Department of Biology, University of Naples Federico II, 80139 Naples, Italy; daniele.deluca@unina.it (D.D.L.); alessandro.derosa.proxy@gmail.com (A.D.R.); 5 Department of Life and Environmental Sciences, University of Cagliari, 09123 Cagliari, Italy; bacchet@unica.it (G.B.); msarigu@unica.it (M.S.)

**Keywords:** Mediterranean Basin, Anthemideae, endemism, niche similarity, morphometrics, image analysis, molecular analysis

## Abstract

**Simple Summary:**

Systematics is the branch of biology that studies the relationships among organisms and their evolution, while taxonomy is the science of classification. In this work, a systematic and taxonomic investigation about three plant species of *Santolina*, commonly known as lavender-cotton, is presented. Two of these species occur exclusively in Corsica and Sardinia, two of the main islands of the Mediterranean Sea, while a third one is a common ornamental plant, known only as cultivated. By integrating several approaches, we find out that the two putative species from Corsica and Sardinia are actually very similar from many points of view. A two-species hypothesis is no longer supported according to our results, so that these plants should be reclassified as a single species. This study demonstrates the importance of integrating different sources of information to produce reliable classifications (i.e. taxonomic hypotheses). In addition, our study is useful to better understand plant evolution in the context of the Mediterranean Basin, one of the world’s biodiversity hotspots.

**Abstract:**

*Santolina* is a plant genus of dwarf aromatic shrubs that includes about 26 species native to the western Mediterranean Basin. In Corsica and Sardinia, two of the main islands of the Mediterranean, *Santolina corsica* (tetraploid) and *S. insularis* (hexaploid) are reported. Along with the cultivated pentaploid *S. chamaecyparissus*, these species form a group of taxa that is hard to distinguish only by morphology. Molecular (using *ITS*, *trnH-psbA*, *trnL-trnF*, *trnQ-rps16*, *rps15-ycf1*, *psbM-trnD*, and *trnS-trnG*), cypsela morpho-colorimetric, morphometric, and niche similarity analyses were conducted to investigate the diversity of plants belonging to this species group. Our results confute the current taxonomic hypothesis and suggest considering *S. corsica* and *S. insularis* as a single species. Moreover, molecular and morphometric results highlight the strong affinity between *S. chamaecyparissus* and the *Santolina* populations endemic to Corsica and Sardinia. Finally, the populations from south-western Sardinia, due to their high differentiation in the studied plastid markers and the different climatic niche with respect to all the other populations, could be considered as an evolutionary significant unit.

## 1. Introduction

With about 30,000 taxa of vascular plants, the Mediterranean biogeographic region is one of the 34 most important mega hot-spots for biodiversity in the world [1,2]. The endemic-vascular plant richness (EVPR) in these hotspots is >2,000 species per 15,000 km^2^, and within these areas at least 10% of narrow endemics occur [2]. One of the causes of the high level of biodiversity in that area lies in its peculiar geological and climatic history during the last 20 million years [3,4]. Indeed, the shift from a sub-tropical to a Mediterranean climate, glaciations, oscillations of the sea level, tectonic movements, and human activity played a crucial role in the diversification of plants triggering speciation processes [5,6,7]. The Tyrrhenian islands (sensu Médail and Quézel [8]) of the central-western Mediterranean Sea (Sicily, Sardinia, Corsica, Tuscan Archipelago, and Balearic Islands), with approximately 6000 plant species, of which 10–12% are endemic [9], are considered one of the 11 macro hot-spots within the Mediterranean region [10]. Corsica (France) and Sardinia (Italy) are two of the largest islands of the Mediterranean Basin and host a great number of endemic vascular plants. Indeed, in Sardinia, 319 out of 2462 taxa are endemic [11], and 316 out of 2781 in Corsica [12]. The study of species that are endemic to both islands allows a better understanding of the evolutionary dynamics in the Mediterranean Basin [13,14,15,16]. In this context, the populations of *Santolina* L. (Asteraceae, Anthemideae) occurring in Corsica and Sardinia are a good case study.

*Santolina* is a genus of dwarf aromatic shrubs that includes about 26 species endemic to the western portion of the Mediterranean Basin [17,18,19]. According to Oberprieler [20], the diversification of this genus from its closest relative started around 10 million years ago. The centre of diversification is probably the Iberian Peninsula, where the highest number of *Santolina* species is reported [21]. *Santolina corsica* Jord. & Fourr. and *S. insularis* (Gennari ex Fiori) Arrigoni, the two species occurring in Corsica and Sardinia, belong to the *S. chamaecyparissus* L. complex, which includes 14 species distributed in the central-western Mediterranean [17]. According to the most recent floras [18,22,23,24], these two taxa and *S. chamaecyparissus* s.str. form an easily distinguishable species group within the *S. chamaecyparissus* complex. All the three species are polyploid and show a distinctive combination of morphological characters: yellow flowers, tomentose stems, and long and tomentose leaves with short and obtuse segments.

*Santolina insularis* is endemic to Sardinia and is hexaploid with 2*n* = 6*x* = 54 chromosomes. *Santolina corsica* is recorded for both islands and is tetraploid with 2*n* = 4*x* = 36 chromosomes [24]. In Sardinia, it is recorded only for Monte Albo, in the central-eastern portion of the island, so that it shows an allopatric distribution with *S. insularis*. *Santolina chamaecyparissus* is a pentaploid sterile unit of unknown origin, and it is only known as a cultivated plant [19]. It is morphologically similar to *S. corsica*/*S. insularis* and shows an irregular karyotype structure [24,25]. Marchi and D’Amato [26] hypothesized that this pentaploid species may have arisen from a cross between a tetraploid and a hexaploid *Santolina*, involving possibly *S. insularis* as one of the putative parents.

The phenotypic differentiation of these three species is not straightforward. When Fiori [27] published the basionym of *S. insularis*, he expressed doubts regarding the taxonomic distinction of the newly described taxon with *S. corsica*. Later, the same author [28] considered these taxa as distinct, although he remarked their morphological affinity. In the same work, Fiori [28] reported several localities for *S. insularis* in continental Italy, but these localities were later confuted by Arrigoni [25], who referred them to naturalized populations of *S. chamaecyparissus*. Chromosome counts published by Marchi and D’Amato [26] and Marchi and collaborators [29] highlighted different ploidy levels for these three species. Accordingly, the current taxonomic circumscription of *S. corsica* and *S. insularis* heavily relies on the different ploidy levels and, secondarily, on qualitative morphological observations [30].

The aim of this study is to test the current two-species taxonomic hypothesis for Sardinia and Corsica by using an integrated approach [31,32], that involves molecular analyses, cypsela morpho-colorimetry, morphometry, and niche similarity tests. *Santolina chamaecyparissus*, traditionally considered as closely related, is also included in the study.

## 2. Materials and Methods

### 2.1. Collection of Material and Data

**Figure 1:**
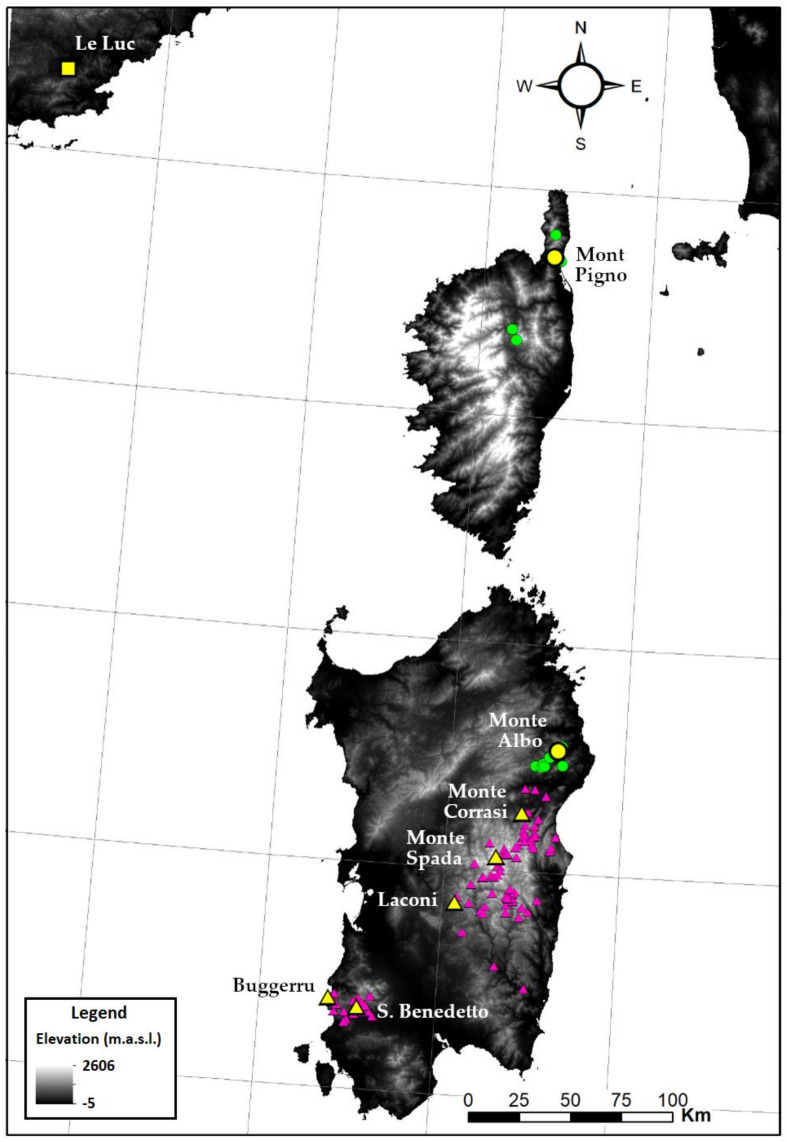
Geographical distribution of *Santolina corsica* (green dots) and *S. insularis* (purple triangles). The sampled populations are in yellow. The only square in southern continental France represents the naturalized sampled population of *S. chamaecyparissus* from Le Luc (Provence-Alpes-Côte d’Azur).

For molecular, cypsela morpho-colorimetric, and morphometric analyses, five populations of *S. insularis*, two populations of *S. corsica*, and one naturalized population of *S. chamaecyparissus* were sampled (Table 1). For morphometric analyses, 20 individuals were collected for each population (nine for *S. chamaecyparissus*, corresponding to the whole naturalized population). Leaf material from 3 out of the 20 individuals was taken, preserved in silica-gel, and was used for molecular analyses. As regards cypsela morpho-colorimetric analysis, cypselae of the Sardinian populations were already available at the Sardinian Germplasm Bank of the University of Cagliari (BG-SAR). Cypselae of the Corsican population of *S. corsica* were sampled in the field, while *S. chamaecyparissus* has been excluded from this analysis since no cypsela was found in the field. For the niche similarity tests, occurrence data from Sardinia were downloaded from Wikiplantbase #Sardegna [33], while occurrence data for Corsica were obtained from literature [27] and personal communications (J.-M. Tison). *Santolina chamaecyparissus* was excluded from the niche analysis, since this species is of unknown origin and its distribution is crucially affected by human cultivation [19]. Distribution of species and sampled populations are summarized in Figure 1.

### 2.2. Molecular Analysis

DNA was extracted using the kit ExgeneTM Plant SV Mini (GeneAll Biotechnology, Singapore). Both nuclear (*ITS* region) and plastid markers (*trnH-psbA*, *trnL-trnF*, *trnQ-rps16*, *rps15-ycf1*, *psbM-trnD*, and *trnS-trnG*) were screened for variability. Amplification was carried out with a Polymerase Chain Reaction (PCR) using the primers reported in Table 2 (amplification conditions for each marker are reported in Table 3). The following reagents were added in 0.2 mL Eppendorf tubes: 12.5 μL of Kodaq 2X PCR MasterMix (Applied Biological Materials, British Columbia, Canada), 1 μL of forward primer 10 μM, 1 μL of reverse primer 10 μM, and the previously extracted DNA (0.5–1.5 μL); then, tubes were filled with distilled water up to the volume of 25 μL. PCR products underwent quality checks and purification. The final product (5 μL) was then diluted with 8 μL of distilled water, and, finally, underwent capillary electrophoresis with a 3130 Genetic Analyzer (Applied Biosystems, Massachusetts, USA).

The electropherograms were edited with the software Sequence Scanner 2 (Applied Biosystems, Massachusetts, USA), while sequences were edited and aligned with the software Bioedit v. 7.2.5 [34] and Clustal W [35]. The sequences were submitted to GenBank (Table S1). The nuclear markers were not further analyzed because of the total absence of variation among sequences. Concerning cpDNA markers, a haplotype network based on the concatenated alignment was built using the software TCS v. 1.21 [36]. Gaps were treated as missing data.

**Table 2:** Primers used for each molecular marker. The first row of each locus is the *forward*, while the second row is the *reverse*.

Marker	Name	Sequence 5′–3′	Reference
*ITS1-5.8S-ITS2*	JK14	GGA GAA GTC GTA ACA AGG TTT CCG	[37]
	JK12	CCA AAC AAC CCG ACT CGT AGA CAG C	
*trnQ-rps16*	trnQ	GCG TGG CCA AGY GGT AAG GC	[38]
	rps16	GTT GCT TTY TAC CAC ATC GTT T	
*trnH-psbA*	psbA	GTT ATG CAT GAA CGT AAT GCT C	[39]
	trnH	CGC GCA TGG TGG ATT CAC AAT CC	
*trnL-trnF*	trnF-IGS-f	GGT TCA AGT CCC TCT ATC CC	[39]
	trnF-IGS-r	ATT TGA ACT GGT GAC ACG AG	
*trnS-trnG*	trnS2-f	CGG TTT TCA AGA CCG GAG CTA TCA A	[40]
	trnG2-r	CAT AAC CTT GAG GTC ACG GGT TCA AAT	
*psbM-trnD*	psbM-f	TTT GAC TGA CTG TTT TTA CGT A	[40]
	trnD-r	CAG AGC ACC GCC CTG TCA AG	
*rps15-ycf1*	rps15-IGSR	GCA ATT CTA AAT GTG AAG TAA G	[41]
	ycf1-IGSR	ATT ATC GAT TAG AAG ATT TAG C	

**Table 3:** Amplification conditions for the studied molecular markers.

Locus	Denaturation (Start)	Denaturation	Annealing	Extension	Extension (End)
			×35		
*ITS1-5.8S-ITS2*	94 °C 5′	94 °C 45″	55 °C 45″	72 °C 1′30″	72 °C 7′
			×35		
*trnQ-rps16*	94 °C 3′	94 °C 30″	53 °C 30″	72 °C 1′	72 °C 3′
			×30		
*trnH-psbA*	95 °C 2′	95 °C 30″	55 °C 30″	72 °C 35″	72° C 1′
			×30		
*trnL-trnF*	95 °C 2′	95 °C 30″	55 °C 30″	72 °C 35″	72 °C 1′
			×35		
*trnS-trnG*	94 °C 3′	94 °C 30″	58 °C 30″	72 °C 1′30″	72 °C 5′
			×35		
*psbM-trnD*	94 °C 3′	94 °C 30″	55 °C 30″	72 °C 1′30″	72 °C 5′
			×35		
*rps15-ycf1*	94 °C 3′	94 °C 30″	55 °C 30″	72 °C 1′30″	72 °C 5′

### 2.3. Cypsela Morpho-Colorimetric Analysis

In order to check for possible differences in external shape, size, and colour of cypselae, a morpho-colorimetric analysis was carried out. This approach is based on image analysis and turned out to be useful to untangle discrimination problems both in cultivated [42,43] and in wild plants [44,45,46]. Digital images of 100 cypselae for each population (99 for *S. insularis* from Monte Spada) were acquired using a flatbed scanner (Epson Perfection V550) with a digital resolution of 1,200 dpi. The scanner was calibrated following the protocol proposed by Shahin and Symons [47]. To avoid interference from environmental light, cypselae were randomly disposed on the scanner tray and covered by a box with white paper and then with a box with black paper. After acquisition, descriptors of cypsela size, shape, and colour features were measured and analyzed using ImageJ v1.52b (http://rsb.info.nih.gov/ij, accessed on 25 November 2021). For each cypsela, 20 colorimetric and 26 morphometric characters were measured using the plugin Particles8 [48]. This plugin was also able to obtain 78 additional morphological parameters by computing the elliptic Fourier descriptors (EFDs). To minimize measurement errors and to optimize the efficiency of shape reconstruction, 20 harmonics were used to define the cypsela boundaries [49,50]. In Figure S1, a graphic explanatory representation of some morphometric variables analyzed by this approach is reported.

After standardization of data, a stepwise Linear Discriminant Analysis (hereafter LDA) was carried out to test the correct classification of groups (putative species and populations). This analysis was performed using the software SPSS 16.0 (Statistical Package for Social Science, IBM Corp., Armonk, NY, USA). Tolerance and F-to-Remove parameters were used by the model to select the best set of variables. Tolerance indicates the proportion of the variance of a factor not accounted by other independent variables, while F-to-remove defines the power of each variable in the model [42]. The performance of the model was tested with a leave-one-out cross-validation procedure. Moreover, for each discriminant function, the Wilks’ λ, the percentage of explained variance, and the standardized canonical discriminant function coefficients (SCDFCs) were calculated. Wilks’ λ is a measure of how well a discriminant function separates cases into groups, while SCDFCs allows comparing and ranking variables measured on different scales. The Box’s M test was used to verify the homogeneity of the covariance matrices of the variables chosen in the stepwise LDA, while the analysis of standardized residuals was performed to verify the homoscedasticity of the variance of variables [51]. Kolmogorov-Smirnov’s test was performed to compare the empirical distribution of the discriminant functions with the relative cumulative distribution function of the reference probability distribution. Levene’s test was performed to assess the equality of variances for the discriminant functions.

### 2.4. Morphometric Analysis

Forty-four morphological characters were measured on dried material (the list of the morphological characters are reported in Table 4). Characters were selected among those used in literature for species discrimination [18,22,23,30]. Depending on the character (Table 4), measurements were made using a digital caliper or ImageJ v.1.52b software. For this latter case, images with a resolution of 1,200 dpi were acquired using a flatbed scanner (EPSON perfection 2480 photo). As regards the degree of tomentosity on leaves and stems (fs_hair, fsl_hair, and ssl_hair in Table 4), images were acquired using a digital camera (Canon PowerShot S45) mounted on a WILD Heerbrugg M420 stereomicroscope. For these variables, a portion of stem and a leaf segment was selected, and the degree of tomentosity was calculated by dividing the surface covered by hair by the total selected surface. After the measurements, specimens have been preserved in PI (acronym follows Thiers’ Index Herbariorum [52]) and were digitized (HD images are available at JACQ Virtual Herbaria: https://www.jacq.org/, accessed on 9 December 2021).

To explore the overall morphological variability, a principal coordinate analyses (PCoA) based on Gower distance [53] was carried out after standardization of variables. The Random Forest (RF) method was used to test the correct classification of a priori established groups according to the current taxonomic hypothesis and to alternative grouping hypotheses derived from other analyses. Random Forest is increasingly being used as a classification method in morphometric analyses [54,55,56], since it is able to manage datasets containing both quantitative and qualitative variables. In addition, it is non-parametric, hard to overtrain, and it can also be used when there is covariation among variables [57,58]. It is an algorithm based on decision trees, where each decision tree works on a bootstrap dataset. At each node of the tree, the algorithm uses a subset of random variables. After the “forest” (it consists of some hundreds of decision trees) is built, each decision tree casts an unweighted vote by assigning each sample to a group. Finally, the membership of a sample to one group rather than another depends on the total number of votes. The algorithm works firstly on a training dataset, and then it repeats the procedure on the remaining subset, called “test set”. Random Forest analysis was carried out in R environment using the package “randomForest” (version 4.6-14, [59]). The function tuneRF was used to calculate the optimal number of variables to use at each node. One hundred iterations of this function were applied, and the suggested optimal number showing the highest frequency was selected. Then, a forest consisting of 800 decision trees was built. The function varImp was used to rank the variables (morphological characters) according to their discriminant power using the metric “mean decrease accuracy” (MDA). We specified the argument TRUE so that the algorithm evaluates the possibility of covariation among variables. For each tested hypothesis, random forest was repeated 10 times with different random “seeds” using the set.seed function. The 10 confusion matrices produced were used to calculate a “mean” confusion matrix, composed of mean percentage values of classification.

Finally, univariate analyses were conducted on all the quantitative variables comparing populations. A Bartlett test was performed to test the homoscedasticity. When *p* > 0.05, ANOVA followed by Tukey-Kramer post hoc test were performed. When *p* < 0.05, Kruskal-Wallis test followed by Wilcoxon–Mann–Whitney with Bonferroni correction were performed.

### 2.5. Niche Analysis

Following the approach published by Broennimann and collaborators [60], we carried out a niche analysis in a multivariate space defined by the climatic conditions in which the taxa occur. We firstly tested the current taxonomic hypothesis, and then we tested two alternative taxonomic hypotheses resulting from cypsela morpho-colorimetric and molecular analyses.

For each taxonomic hypothesis, we calculated the niche overlap between groups using Schoener’s D overlap [61], whose values range from 0 (no overlap) to 1 (full overlap). Then, we used the similarity test to evaluate whether niches are more or less similar to each other than predicted by chance. The observed niche overlap was compared with the overlap measured between the niche of one group and the niche obtained by randomly sampling occurrence points in a background area of the other group. This randomization was repeated 100 times. To test whether our results are robust to different selections of the background, we defined three background areas using a 5, 10, and 15 km buffer zone around the occurrences. The analyses were conducted in R (R Core Team, 2019) using the “ecospat” package [62].

## 3. Results

### 3.1. Molecular Analysis

**Figure 2:**
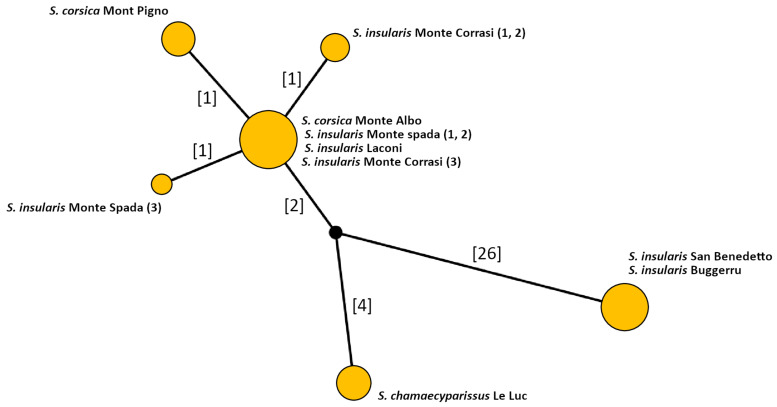
Haplotype network based on the cpDNA markers listed in Table 2. The yellow circles represent groups of *Santolina* individuals sharing the same haplotype, while the numbers in square brackets are the number of mutational steps among different haplotypes. The black circle is a non-sampled haplotype, hypothesized by the algorithm, connecting the three haplotype groups.

The nuclear marker *ITS1-5,8s-ITS2* (861 bp) did not provide any informative site and was therefore excluded from further inquiry (alignment is provided in Supplementary Materials). Concerning cpDNA data, the markers *trnH-psbA* and *trnQ-rps16* show the highest number of informative sites, followed by *rps15-ycf1* and *psbM-trnD* (Table 5). Conversely, the marker *trnF-trnL* shows only one informative site. The concatenated matrix is 3,403 bp long and the number of informative sites is 34 (1%). Three haplotype groups are evident. The first group includes the two populations of *S. insularis* from south-eastern Sardinia (Buggerru and San Benedetto), which share the same haplotype. The second group includes *S. chamaecyparissus* only, while the third group includes all the remaining accessions, subdivided in four haplotypes. In particular, *S. corsica* from Mont Pigno belongs to a distinct haplotype, as well as two out of three individuals of *S. insularis* from Monte Corrasi and one individual of *S. insularis* from Monte Spada (Figure 2).

### 3.2. Cypsela Morpho-Colorimetric Analysis

The LDA selected 14 variables (Table S2) for their discriminant power. The EFDs and the colorimetric parameters were not considered useful by the algorithm and were discarded. In Table 6, the confusion matrix of the current taxonomic hypothesis is reported. Overall, the percentage of correct classification is 77.9%. An additional LDA was carried out considering all the populations as groups (the confusion matrix is reported in Table 7). In this case, the percentage of correct classification is obviously lower (51.9%). The three populations of *S. insularis* from central-eastern Sardinia show values of correct classification ranging between 53% and 66%. They tend to confuse each other, but they are scarcely confused with all the other populations. Similarly, all the other populations tend to confuse each other, but are scarcely confused with the populations of *S. insularis* from central-eastern Sardinia. This slight distinction allows the detection of two groups of affinity: the first one is composed of the populations of *S. insularis* from central-eastern Sardinia (Laconi, Monte Spada, and Monte Corrasi), and the second one composed of all the remaining populations of *S. insularis* and *S. corsica*. The dispersion of the standardized residuals tested by Levene’s test, and the normal probability plot (P-P) comparing expected and observed cumulative probabilities are reported in Figure S2.

### 3.3. Morphometric Analysis

**Figure 3:**
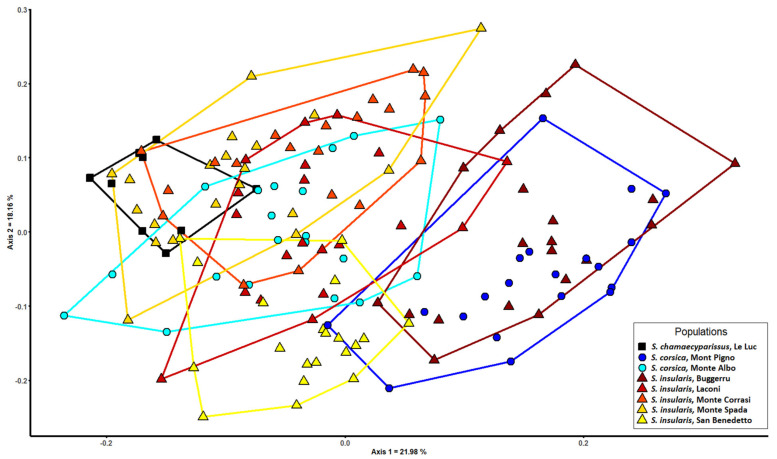
Morphometric analysis of *Santolina chamaecyparissus*, *S. corsica*, and *S. insularis*. Scatter plot of the first two axes of the PCoA based on Gower distance.

Two slightly separated groups can be observed in the PCoA (Figure 3): the first one is composed of *S. corsica* from Mont Pigno (type locality) and *S. insularis* from Buggerru, while the second one consists of all the remaining populations. In Table 8, the result of the Random Forest analysis testing the current taxonomic hypothesis is reported. *Santolina corsica* shows the lowest mean value of correct classification (46%), and it is largely misclassified as *S. insularis*. No individual samples of *S. insularis* or *S. corsica* are misclassified as *S. chamaecyparissus*.

Random forest was also used to test alternative taxonomic hypotheses that can be deduced by the information derived from PCoA, from cypsela morpho-colorimetric analysis, and from molecular analysis. According to PCoA, we split populations from Corsica and Sardinia into two groups (Table 9). *Santolina chamaecyparissus* is misclassified in 10% of cases with “Group 2”. However, no individual of other groups is misclassified with *S. chamaecyparissus*. The other two groups show higher values of correct classification (82% and 97%), when compared to the groups tested in the current taxonomic hypothesis (Table 8).

Furthermore, the two groups of affinity highlighted by the cypsela morpho-colorimetric analysis show similar correct mean classification rates (Table 10): 83% for “Group A” (populations of *S. insularis* from central-eastern Sardinia) and 94% for “Group B” (all the remaining populations of *S. insularis* and *S. corsica*). *Santolina chamaecyparissus* is misclassified in 26% of cases with populations from central-eastern Sardinia albeit, also in this case, no individual of other groups is misclassified with it.

Finally, the results of Random Forest analysis applied to the three haplotype groups detected by molecular analysis are reported in Table 11. The group composed of the populations of *S. corsica* and *S. insularis* from central-eastern Sardinia shows high mean correct classification rates (99%), while the group composed of the two populations from south-eastern Sardinia show considerably lower values (57%). *Santolina chamaecyparissus* is classified correctly in 78% of cases on average, and no individual of other groups is misclassified with it.

In Table 12, the mean (± standard deviation) values of quantitative morphological characters for each *Santolina* population are reported. All populations differ significantly from each other by 2–14 characters. The minimum number of characters is found by comparing Monte Corrasi with Laconi and Monte Spada populations. The maximum number of characters is found by comparing *S. chamaecyparissus* with Mont Pigno (Table 12 and Table S3).

### 3.4. Niche Analysis

According to the current taxonomic hypothesis, the niche overlap between the two putative species ranges from 0.26 to 0.36, and the similarity test shows that the climatic conditions of *S. corsica* are more similar to those of *S. insularis* in the three background areas. According to the alternative grouping derived from molecular results, the niche overlap ranges from 0.03 to 0.05, and the similarity test was not significant. Finally, according to the alternative grouping derived from the cypsela morpho-colorimetric analysis, the niche overlap ranges from 0.09 to 0.25, and the similarity test indicated that the climatic conditions of the group composed of *S. insularis* of south-western Sardinia plus *S. corsica* are significantly more similar than vice versa (Table 13).

## 4. Discussion

The current circumscription of *S. corsica* and *S. insularis* is not supported on morphological grounds. This result is in accordance with Angiolini and Bacchetta [63], who stated that it is impossible to distinguish the two taxa only by morphological features. Conversely, our results are in contrast with Arrigoni and collaborators [30] and Arrigoni [22], who consider the distinction between the two putative species as sufficiently clear. According to their qualitative morphological observations, the main differences would lie in the colour of the flowers, the colour of the anthers, the peduncle under the floral head (more widened in *S. insularis*), and the length of the segments of the sterile stem leaves. In the field, we detected no difference in the colour of the flowers and anthers, which are as yellow as the majority of the other *Santolina* species [18,22,23]. Moreover, the width of the floral head peduncle is an extremely variable character (also within the same individual), so that it was preliminarily discarded from the list of morphological variables studied. As regards the length of the leaf segments, according to Arrigoni and collaborators [28] and Arrigoni [48], they should be 1–3 mm long in *S. corsica*, and 2–5 mm long in *S. insularis*. We did not detect significant differences for this character among populations (with the exception of the comparison Buggerru vs. Mont Pigno), and all the populations show the same range of variation (fsl_seg_length and ssl_seg_length in Table 12).

According to molecular analysis, all the studied populations share identical sequences of the *ITS* region, suggesting close phylogenetic relationships. Despite this, based on plastid markers, three haplotype groups can be detected: (1) *S. chamaecyparissus*, (2) the populations from Corsica and central-eastern Sardinia, and (3) the two populations from southern-western Sardinia (Figure 2). These latter populations are separated from others by Campidano graben, the greatest plain valley in Sardinia [63]. This plain was formed during the middle Pliocene and was submerged several times by marine transgression events [64,65] causing isolation. In addition, according to niche similarity tests, it seems that the Sardinian south-western populations occur in different climatic conditions with respect to all other populations from Corsica and Sardinia. The different climatic niche could reflect a potential adaptive differentiation, that allow us to define these populations as a full Evolutionary Significant Unit (full ESU). A full ESU, as defined by Guia and Saitoh [66] based on a concept previously theorized by Ryder [67], is a population showing both genetic and adaptive variation. In addition, an ESU represents a unit with a strong evolutionary potential, relevant for conservation purposes [68]. However, from a morphological point of view, the two populations from southern-western Sardinia are far from similar (Figure 3). The population from Buggerru is morphologically much more similar to the population from Mont Pigno (Corsica, type locality of *S. corsica*), whereas that from San Benedetto (type locality of *S. insularis*) approaches all the remaining populations.

The cypsela morpho-colorimetric analysis points to a different grouping as well. Indeed, the three populations of *S. insularis* from central-eastern Sardinia (Laconi, Monte Spada, and Monte Corrasi) show a certain affinity, and are scarcely misclassified with the remaining populations. However, according to the results of the LDA, these three populations are only slightly differentiated from the remaining ones, and the values obtained by the LDA were more related to the cypsela morphology than colour (Table S2). The overall morphological homogeneity is in accordance with Briquet [69], who stated that the cypsela morphology is very similar in the whole genus *Santolina*.

Finally, climatic niches are overlapping following both the current taxonomic hypothesis and the grouping suggested by the cypsela morpho-colorimetric analysis.

Each different method used in our integrated taxonomic approach tends to group the studied populations in different ways. In any case, the current taxonomic circumscription of *S. corsica* and *S. insularis* is no longer supported. According to Giacò and collaborators [24], despite the different ploidy level, these two species show a very similar karyotype structure. Based on all this evidence, we think that the best option is to consider the tetraploid and hexaploid populations of *Santolina* from Corsica and Sardinia as a single polymorphic species, showing two cytotypes. Due to nomenclatural priority, the name that has to be applied is *Santolina corsica* Jord. & Fourr., so that the name *Santolina insularis* (Gennari ex Fiori) Arrigoni becomes a heterotypic synonym.

In literature, other cases of species endemic to Corsica and Sardinia showing multiple cytotypes are reported. For instance, *Thymus herba-barona* Loisel. (Lamiaceae) is a polymorphic species endemic to Corsica, Sardinia, and Mallorca, showing three different ploidy levels. A population genetic study [14] highlighted that, despite the high morphological and genetic variability among populations, there is no phylogeographic discontinuity among islands, so that the cytogenetic differences are not sufficient to support the distinction of three different taxa, as previously proposed. Another similar case concerns *Borago pygmaea* (DC.) Chater & Greuter (Boraginaceae), a species endemic to Corsica, Sardinia, and Capraia (a small island between Corsica and continental Italy), which shows both tetraploid and hexaploid ploidy levels [13]. Finally, another case of a single species showing two cytotypes can be found within the *S. chamaecyparissus* complex: *S. villosa* Mill. is a species endemic to continental Spain, which includes both tetraploid and hexaploid populations [24].

Our molecular and morphometric results also confirm that *S. chamaecyparissus* may be a species arisen from a cross between a tetraploid and a hexaploid *Santolina*, similar or identical to at least one of those growing in Sardinia and Corsica, as previously hypothesized by Marchi and D’Amato [26] and Giacò and collaborators [24]. In particular, morphometric analysis suggests that *S. chamaecyparissus* is very similar to the populations from central-eastern Sardinia (Figure 3). Despite this, when considered as a distinct a priori group in Random Forest tests, no individual of other populations is misclassified as *S. chamaecyparissus*. Accordingly, on taxonomic grounds, *S. chamaecyparissus* can be considered as a distinct species, thanks to its karyological and morphological peculiarities. For instance, it shows a lower number of leaf segments (see fsl_n_seg and ssl_n_seg in Table 12 and Table S3), and the tubular portion of the flowers is longer (flower_length in Table 12 and Table S3), if compared with all the other studied populations. If *S. chamaecyparissus* ever existed as a wild species in the past, we may assume that it possibly occurred in Sardinia, where both putative tetraploid and hexaploid wild progenitors can be found. Alternatively, *S. chamaecyparissus* may also be a species of full anthropogenic origin, possibly also involving other *Santolina* species as progenitors. In this context, we highlight that this plant was already widely known at the time of Pliny the Elder (AD 23–79; Naturalis Historia XXIV: “Chamaecyparissos herba ex vino pota contra venena serpentium omnium scorpionumque pollet” [the plant “Chamaecyparissos”, drunk with wine, is effective against the venoms of every snake and scorpion]). However, more studies are needed to detect the origin of this pentaploid species, as well as that of the Sardinian and Corsican tetraploid and hexaploid cytotypes in a complex mostly composed of diploid species [24]. To address these questions, an integrative taxonomic and systematic study extended to the whole *S. chamaecyparissus* complex is ongoing.

## 5. Conclusions

This work contributed to a better understanding of plant diversity in a complex evolutionary context such as the insular system in the Mediterranean region, which hosts a high number of endemic species. Our integrated taxonomic approach highlighted the impossibility of consistently separating *S. insularis* from *S. corsica*, which have to be considered as just two cytotypes of the same species, according to our data. Indeed, the populations of *Santolina* from Corsica and Sardinia are polymorphic, but the morphological differences are not related neither with the ploidy level nor with cpDNA variation, albeit the identical *ITS* region sequences suggest a strong relatedness of these populations. Even without considering ploidy levels as relevant, cpDNA variation alone is not sufficient to justify a different taxonomic treatment for the populations from southern Sardinia. However, thanks to their genetic distance, coupled with different climatic niches, we propose treating these populations as an Evolutionary Significant Unit (ESU). Finally, our results highlighted a strong affinity of the pentaploid cultivated *S. chamaecyparissus* with the populations from Corsica and Sardinia. The taxonomic consequences of our study are the following:

***Santolina corsica*** Jord. and Fourr., Icon. Fl. Eur. 2: 9. 1869 ≡ *Santolina chamaecyparissus* var. *incana* subvar. *corsica* (Jord. and Fourr.) Rouy, Fl. France 8: 223. 1903 ≡ *Santolina chamaecyparissus* var. *corsica* (Jord. and Fourr.) Fiori, Nuov. Fl. Italia 2: 659. 1927–Lectotype (designated by Giacò et al. in Taxon 70(1): 194. 2021): [illustration] “*Santolina corsica* Jord. et Fourr.” in Jordan and Fourreau, Icon. Fl. Eur. 2: t. 227. 1869.

= *Santolina chamaecyparissus* var. *pectinata* f. *insularis* Gennari ex Fiori in Fiori et al., Fl. Italia 3(1): 270. 1903 ≡ *Santolina chamaecyparissus* subsp. *insularis* (Gennari ex Fiori) Yeo in Bot. J. Linn. Soc. 70: 18. 1975 ≡ *Santolina insularis* (Gennari ex Fiori) Arrigoni in Webbia 34(1): 263. 1979–Lectotype (designated by Arrigoni in Boll. Soc. Sarda Sci. Nat. 21: 338. 1982, as “holotype”): Italy, Sardinia, Monti d’Iglesias a S. Benedetto, s.d., *P. Gennari s.n.* (FI barcode FI002787!).

## Figures and Tables

**Table 1:** Sampled populations with voucher information.

Species	Population	Vouchers
*S. chamaecyparissus*	France, Provence-Alpes-Côte d’Azur, Le Luc [WGS84: 43.354166 N, 6.412222 E]	*A. Giacò and L. Peruzzi*, 30 June 2020, PI 034970–034974
*S. corsica*	France, Corsica, Mont Pigno [WGS84: 42.7066667 N, 9.407777 E], type locality	*A. Giacò and L. Peruzzi*, 7 July 2020, PI 036636–036647
*S. corsica*	Italy, Sardinia, Monte Albo [WGS84: 40.537853 N, 9.615131 E]	*G. Calvia* et al., 19 June 2020, PI 036122–036136
*S. insularis*	Italy, Sardinia, Buggerru [WGS84: 39.393611 N, 8.391666 E]	*G. Bacchetta* et al., 14 June 2020, PI 036613–036625
*S. insularis*	Italy, Sardinia, San Benedetto (Iglesias) [WGS84: 39.360311 N, 8.558333 E], type locality	*G. Bacchetta* et al., 14 June 2020, PI 036068–036085
*S. insularis*	Italy, Sardinia, Laconi [WGS84: 39.847483 N, 9.071944 E]	*G. Bacchetta* et al., 15 June 2020, PI 036052–036067
*S. insularis*	Italy, Sardinia, Monte Spada [WGS84: 40.058586 N, 9.293333 E]	*G. Bacchetta* et al., 14 June 2020, PI 036106–036121
*S. insularis*	Italy, Sardinia, Monte Corrasi [WGS84: 40.256878 N, 9.426253 E]	*G. Bacchetta* et al., 14 June 2020, PI 036648–036663

**Table 4:** Morphometric characters and their description. QC = quantitative continuous, QD = quantitative discrete, CN = nominal, CB = bimodal, and CO = ordered factor.

Code	Description of the Character	Type	Tool
Vegetative Parts			
fs_length	Length of the fertile stem (cm)	QC	Ruler
br_ratio	Ratio between the highest ramification of the fertile stem and fs_length	QC	Ruler
dist_cap	Distance between the highest leaf on the stem and the floral head (mm)	QC	Caliper
fs_n_br	Number of branches of the fertile stem	QD	
br_type	Type of branch (no branch/parallel/or erect-patent)	CN	
fs_n_nodes	Number of nodes of the fertile stem	QD	
fs_node_length	Length of a random selected node of the fertile stem (mm)	QC	Caliper
fs_sterile_like	Fertile stem morphologically similar to sterile stems (Yes/No)	CB	
ss_length	Length of the sterile stem (cm)	QC	Ruler
ss_n_nodes	Number of nodes of the sterile stem	QD	
ss_node_length	Length of a random selected node of the sterile stem (mm)	QC	Caliper
ss_hair	Tomentosity of the sterile stem (hairless/slightly pubescent/pubescent/hairy/densely hairy)	CO	ImageJ
fs_hair	Degree of tomentosity of the fertile stem (%)	QC	ImageJ
fasc_type	Axillary leaves of the fertile stem (absent/fasciculate/ramified)	CN	
fsl_n_seg	Number of segments on the fertile stem leaf (the longest)	QD	
ssl_n_seg	Number of segments on the sterile stem leaf (the longest)	QD	
ssl_length	Length of the sterile stem leaf (mm)	QC	ImageJ
ssl_width	Width of the sterile stem leaf (mm)	QC	ImageJ
ssl_petiole_length	Length of the petiole of the sterile stem leaf (mm)	QC	ImageJ
ssl_seg_length	Length of the segment of the sterile stem leaf (mm)	QC	ImageJ
ssl_seg_width	Width of the segment of the sterile stem leaf (mm)	QC	ImageJ
ssl_seg_dist	Distance between the segments of the sterile stem leaf (mm)	QC	ImageJ
fsl_length	Length of the fertile stem leaf (mm)	QC	ImageJ
fsl_width	Width of the fertile stem leaf (mm)	QC	ImageJ
fsl_petiole_length	Length of the petiole of the fertile stem leaf (mm)	QC	ImageJ
fsl_seg_length	Length of the segment of the fertile stem leaf (mm)	QC	ImageJ
fsl_seg_width	Width of the segment of the fertile stem leaf (mm)	QC	ImageJ
fsl_seg_dist	Distance between the segments of the fertile stem leaf (mm)	QC	ImageJ
ssl_hair	Degree of tomentosity of the sterile stem leaf segment (%)	QC	ImageJ
fsl_hair	Degree of tomentosity of the fertile stem leaf segment (%)	QC	ImageJ
**Floral head**			
cap_diam	Diameter of the floral head involucre (mm)	QC	Caliper
flowers_type	The flowers totally cover the involucre (Yes/No)	CB	
flower_length	Length of the floral tube (mm)	QC	ImageJ
flower_tooth_length	Length of the floral tooth (mm)	QC	ImageJ
sq_ext_length	Length of the external involucral bract (mm)	QC	ImageJ
sq_ext_width	Width of the external involucral bract (mm)	QC	ImageJ
sq_int_length	Length of the internal involucral bract (mm)	QC	ImageJ
sq_int_width	Width of the internal involucral bract (mm)	QC	ImageJ
sq_if_length	Length of the inter-floral bract (mm)	QC	ImageJ
sq_if_width	Width of the inter-floral bract (mm)	QC	ImageJ
sq_if_type	Tip of the inter-floral bract (rounded/truncate)	CB	
sq_if_n_hair	Tomentosity of the inter-floral bract (hairless/slightly pubescent/pubescent/hairy/densely hairy)	CO	ImageJ
sq_ext_hair	Tomentosity of the external involucral bract (hairless/only on the margin/everywhere)	CO	ImageJ
sq_int_hair	Tomentosity of the internal involucral bract (hairless/only on the margin/everywhere)	CO	ImageJ

**Table 5:** Length (bp), consensus length (bp), and number of informative sites of the studied markers.

Markers	Length (bp)	Consensus Length (bp)	Informative Sites
*psbM-trnD*	708–716	717	5
*rps15-ycf1*	495–510	517	7
*trnF-trnL*	381	381	1
*trnH-psbA*	422–457	458	9
*trnQ-rps16*	885–895	905	10
*trnS-trnG*	420–425	425	2
Concatenated matrix	---	3403	34

**Table 6:** Cypsela morpho-colorimetric analysis of *Santolina corsica* and *S. insularis*. Confusion matrix of the LDA analysis applied comparing the current taxonomic hypothesis. Values are percentages.

Species	*S. corsica*	*S. insularis*
*S. corsica*	54	46
*S. insularis*	12.4	87.6

**Table 7:** Cypsela morpho-colorimetric analysis of all the studied *Santolina* populations. Confusion matrix of the LDA analysis applied comparing each population of *S. corsica* and *S. insularis*.

Populations	*S. corsica* Monte Albo	*S. corsica* Mont Pigno	*S. insularis* San Benedetto	*S. insularis* Buggerru	*S. insularis* Monte Spada	*S. insularis* Monte Corrasi	*S. insularis* Laconi
*S. corsica* Monte Albo	53	19	15	10	2	1	0
*S. corsica* Mont Pigno	20	56	13	11	0	0	0
*S. insularis* San Benedetto	19	23	24	33	1	0	0
*S. insularis* Buggerru	12	17	22	48	1	0	0
*S. insularis* Monte Spada	0	0	2	0	67	20	11
*S. insularis* Monte Corrasi	0	0	0	0	23	63	14
*S. insularis* Laconi	0	0	0	0	26	21	53

**Table 8:** Morphometric analysis of *Santolina chamaecyparissus*, *S. corsica*, and *S. insularis*. Results of the Random Forest analysis applied to the current taxonomic hypothesis. Values are percentages.

Species	*S. chamaecyparissus*	*S. corsica*	*S. insularis*
*S. chamaecyparissus*	92	0	8
*S. corsica*	0	46	54
*S. insularis*	0	4	96

**Table 9:** Results of the Random Forest analysis applied to the groups of Corsican and Sardinian *Santolina* populations detected by the PCoA. Group 1 is composed of *S. corsica* from Mont Pigno (type locality) and *S. insularis* from Buggerru. Group 2 is composed of all the remaining populations (including the type locality of *S. insularis*). Values are percentages.

Groups	*S. chamaecyparissus*	Group 1	Group 2
*S. chamaecyparissus*	90	0	10
Group 1	0	82	18
Group 2	0	3	97

**Table 10:** Results of the Random Forest analysis applied to the two *Santolina* groups of affinity highlighted by the cypsela morpho-colorimetric analysis. Group A is composed of the populations of *S. insularis* from central-eastern Sardinia (Laconi, Monte Spada, and Monte Corrasi). Group B is composed of all the remaining populations of *S. insularis* and *S. corsica*, including type localities of both names. Values are percentages.

Groups	*S. chamaecyparissus*	Group A	Group B
*S. chamaecyparissus*	74	26	0
Group A	0	83	17
Group B	0	6	94

**Table 11:** Results of the Random Forest analysis applied to the three *Santolina* haplotype groups detected by the molecular analysis. Group *X* is composed of the populations of *S. insularis* from Buggerru and San Benedetto (the type locality). Group *Y* is composed of the populations from Mont Pigno (Corsica, type locality) and by the remaining populations of *S. corsica* and *S. insularis* from Sardinia. Values are percentages.

Groups	*S. chamaecyparissus*	Group *X*	Group *Y*
*S. chamaecyparissus*	78	0	22
Group *X*	0	57	43
Group *Y*	0	1	99

**Table 12:** Mean (± standard deviation) values quantitative morphological characters for each *Santolina* population. Character codes follow Table 4.

Character	*S. chamaecyparissus* Le Luc	*S. corsica* Monte Albo	*S. corsica* Mont Pigno	*S. insularis* San Benedetto	*S. insularis* Buggerru	*S. insularis* Monte Spada	*S. insularis* Monte Corrasi	*S. insularis* Laconi
fs_length (cm)	16.7 (± 2.6)	15.7 (± 3.5)	12.6 (± 3.9)	20.4 (± 5.2)	11.3 (± 4.4)	15.7 (± 4.9)	14.3 (± 4.7)	16.4 (± 3.8)
br_ratio	0.1 (± 0.1)	0.2 (± 0.3)	0.6 (± 0.2)	0.6 (± 0.3)	0.7 (± 0.3)	0.1 (± 0.2)	0.1 (± 0.2)	0.3 (± 0.3)
dist_cap_lf (mm)	41.9 (± 11.6)	24.7 (± 12.4)	20.4 (± 7.3)	34.0 (± 13.8)	11.5 (± 6.4)	36.4 (± 22.5)	35.9 (± 19.2)	27.5 (± 11.6)
fs_n_br	1.0 (± 1.2)	0.7 (± 1.4)	4.0 (± 2.9)	4.0 (± 2.8)	5.1 (± 5.3)	1.0 (± 1.6)	0.4 (± 0.7)	1.5 (± 1.6)
fs_n_nodes	12.9 (± 2.5)	14.8 (± 2.5)	15.0 (± 2.4)	17.3 (± 3.9)	19.9 (± 6.0)	14.4 (± 2.9)	11.5 (± 2.2)	16.3 (± 3.5)
fs_node_length (mm)	13.7 (± 7.1)	9.8 (± 4.1)	8.6 (± 4.2)	12.0 (± 6.4)	6.2 (± 3.5)	7.5 (± 3.2)	9.5 (± 4.9)	9.1 (± 5.6)
ss_length (cm)	10.4 (± 2.8)	9.3 (± 2.2)	6.7 (± 3.6)	11.9 (± 3.8)	5.9 (± 3.2)	10.3 (± 3.7)	7.1 (± 3.2)	10.9 (± 2.9)
ss_n_nodes	16.0 (± 3.2)	17.1 (± 3.2)	15.4 (± 3.1)	18.4 (± 3.1)	16.1 (± 5.2)	16.5 (± 3.3)	12.7 (± 2.8)	17.1 (± 4.8)
ss_node_length (mm)	6.8 (± 2.3)	6.9 (± 2.2)	5.3 (± 2.2)	6.9 (± 2.0)	4.5 (± 2.6)	7.0 (± 3.4)	6.5 (± 3.7)	8.8 (± 3.7)
fs_hair	0.2 (± 0.1)	0.3 (± 0.2)	0.5 (± 0.1)	0.4 (± 0.2)	0.7 (± 0.2)	0.3 (± 0.1)	0.3 (± 0.1)	0.3 (± 0.1)
fsl_n_seg	14.3 (± 3.5)	59.7 (± 17.3)	90.2 (± 28.6)	82.3 (± 15.4)	105.2 (± 32.1)	48.8 (± 9.2)	52.4 (± 16.4)	48.7 (± 15.0)
ssl_n_seg	45.6 (± 6.4)	102.9 (± 20.9)	135.9 (± 37.7)	109.5 (± 18.7)	121.6 (± 31.3)	72.5 (± 12.0)	83.7 (± 14.8)	73.5 (± 16.8)
ssl_length (mm)	23.0 (± 3.4)	36.5 (± 10.4)	33.1 (± 9.3)	38.5 (± 8.8)	24.9 (± 8.3)	23.9 (± 3.5)	26.2 (± 6.8)	25.1 (± 4.5)
ssl_width (mm)	0.9 (± 0.2)	0.8 (± 0.1)	0.8 (± 0.2)	0.8 (± 0.2)	0.8 (± 0.2)	0.9 (± 0.2)	0.8 (± 0.2)	0.8 (± 0.2)
ssl_petiole_length (mm)	3.0 (± 0.8)	4.3 (± 2.2)	3.2 (± 1.6)	4.5 (± 1.8)	3.3 (± 1.5)	2.2 (± 1.0)	2.9 (± 1.0)	3.2 (± 1.8)
ssl_seg_length (mm)	1.5 (± 0.2)	1.2 (± 0.6)	1.5 (± 0.3)	1.4 (± 0.2)	1.0 (± 0.4)	1.3 (± 8.3)	1.3 (± 0.3)	1.3 (± 0.4)
ssl_seg_width (mm)	0.8 (± 0.1)	0.6 (± 0.1)	0.4 (± 0.1)	0.6 (± 0.1)	0.6 (± 0.1)	0.6 (± 0.1)	0.6 (± 0.1)	0.6 (± 0.1)
ssl_seg_dist (mm)	0.8 (± 0.2)	0.9 (± 0.4)	0.9 (± 0.4)	0.7 (± 0.4)	0.4 (± 0.3)	0.8 (± 0.3)	0.9 (± 0.4)	0.7 (± 0.3)
fsl_length (mm)	13.6 (± 2.2)	21.8 (± 8.2)	22.4 (± 5.5)	31.1 (± 8.5)	21.8 (± 8.7)	16.3 (± 4.1)	16.1 (± 4.5)	15.8 (± 5.3)
fsl_width (mm)	1.0 (± 0.2)	0.8 (± 0.4)	0.7 (± 0.2)	0.7 (± 0.1)	0.8 (± 0.2)	0.9 (± 0.2)	1.1 (± 0.7)	0.8 (± 0.2)
fsl_petiole_length (mm)	5.4 (± 1.2)	4.0 (± 1.6)	2.3 (± 1.0)	4.8 (± 1.5)	2.5 (± 1.1)	2.9 (± 1.2)	2.4 (± 1.2)	2.9 (± 1.7)
fsl_seg_length (mm)	1.2 (± 0.2)	0.7 (± 0.3)	1.3 (± 0.4)	1.1 (± 0.3)	0.8 (± 0.2)	1.1 (± 0.4)	0.9 (± 0.2)	1.0 (± 0.4)
fsl_seg_width (mm)	0.6 (± 0.1)	0.5 (± 0.1)	0.4 (± 0.1)	0.6 (± 0.1)	0.6 (± 0.2)	0.6 (± 0.3)	0.5 (± 0.1)	0.5 (± 0.2)
fsl_seg_dist (mm)	0.4 (± 0.3)	0.7 (± 0.4)	0.7 (± 0.2)	0.6 (± 0.3)	0.4 (± 0.3)	0.3 (± 0.3)	0.6 (± 0.3)	0.3 (± 0.3)
ssl_hair	0.9 (± 0.1)	0.7 (± 0.2)	0.8 (± 0.1)	0.8 (± 0.1)	0.8 (± 0.2)	0.7 (± 0.2)	0.9 (± 0.1)	0.6 (± 0.2)
fsl_hair	0.4 (± 0.1)	0.4 (± 0.2)	0.7 (± 0.2)	0.5 (± 0.1)	0.6 (± 0.2)	0.2 (± 0.1)	0.5 (± 0.2)	0.3 (± 0.1)
cap_diam (mm)	7.0 (± 0.4)	7.1 (± 1.6)	5.1 (± 0.7)	6.8 (± 1.1)	5.6 (± 0.8)	6.6 (± 1.1)	6.5 (± 0.9)	6.8 (± 1.3)
flower_length (mm)	4.3 (± 0.3)	3.3 (± 0.4)	2.8 (± 0.3)	3.3 (± 0.4)	3.1 (± 3.1)	3.3 (± 0.4)	3.5 (± 0.3)	2.9 (± 0.5)
flower_tooth_length (mm)	0.6 (± 0.1)	0.8 (± 0.1)	0.7 (± 0.1)	0.7 (± 0.1)	0.6 (± 0.1)	0.7 (± 0.1)	0.8 (± 0.1)	0.7 (± 0.2)
sq_ext_length (mm)	3.3 (± 0.4)	3.2 (± 0.6)	2.9 (± 0.4)	3.6 (± 0.5)	3.3 (± 0.5)	3.6 (± 0.6)	3.2 (± 0.4)	3.3 (± 0.6)
sq_ext_width (mm)	1.6 (± 0.2)	1.3 (± 0.2)	1.2 (± 0.2)	1.3 (± 0.3)	1.3 (± 0.3)	1.4 (± 0.2)	1.3 (± 0.2)	1.3 (± 0.3)
sq_int_length (mm)	4.1 (± 0.3)	3.3 (± 0.4)	2.9 (± 0.3)	3.5 (± 0.5)	3.4 (± 0.4)	4.0 (± 0.5)	3.2 (± 0.6)	3.3 (± 0.5)
sq_int_width (mm)	1.7 (± 0.2)	1.4 (± 0.2)	1.2 (± 0.1)	1.5 (± 0.2)	1.3 (± 0.2)	1.5 (± 0.4)	1.4 (± 0.2)	1.4 (± 0.3)
sq_if_length (mm)	3.7 (± 0.4)	3.4 (± 0.5)	3.1 (± 0.4)	3.3 (± 0.3)	2.9 (± 0.3)	3.4 (± 0.5)	3.9 (± 0.5)	3.1 (± 0.3)
sq_if_width (mm)	1.2 (± 0.2)	1.0 (± 0.2)	0.9 (± 0.2)	1.0 (± 0.2)	1.0 (± 0.2)	1.1 (± 0.2)	1.0 (± 0.3)	1.0 (± 0.2)

**Table 13:** Results of niche overlap and niche similarity tests for the current taxonomic hypothesis and two alternative groupings in *Santolina*. In the alternative grouping derived from cypsela morpho-colorimetric results, “Group A” is composed of the populations of *S. insularis* from central-eastern Sardinia, while “Group B” is composed of *S. corsica* and the populations from south-western Sardinia. In the alternative grouping derived from molecular results, Group *x* is composed of populations from south-western Sardinia, while Group *y* is composed of the remaining populations. Backgrounds are defined by applying 5, 10, and 15 km buffer zones around the occurrence points. Significant results are indicated by ‘less’ for significant divergence or ‘more’ for significant similarity between tests. “ns” (not significant) when *p* > 0.05.

Current Taxonomic Hypothesis			
Background	Niche overlap	*S. corsica* vs *S. insularis*	*S. insularis* vs *S. corsica*
5 km	0.34	more	ns
10 km	0.36	more	ns
15 km	0.26	more	ns
**“Cypsela Morpho-Colorimetric” Grouping**			
Background	Niche overlap	Group A vs Group B	Group B vs Group A
5 km	0.09	ns	more
10 km	0.16	ns	more
15 km	0.25	ns	more
**“Molecular” Grouping**			
Background	Niche overlap	Group *x* vs Group *y*	Group *y* vs Group *x*
5 km	0.04	ns	ns
10 km	0.03	ns	ns
15 km	0.05	ns	ns

## Data Availability

Not applicable.

## Supplementary Materials

The following materials are available online at https://www.mdpi.com/article/10.3390/biology11030356/s1, Table S1: GenBank accession numbers, Table S2: variables used by the LDA in the cypsela morpho-colorimetric analysis, Table S3: morphological characters that are significantly different among *Santolina* populations, Figure S1: graphic explanatory representation of some morphometric variables used in the cypsela morpho-colorimetric approach, Figure S2: residuals plot (a) and normal probability plot (b), “alignments.zip”: alignments (in FASTA format).
