# Supplementary material for: An Integrated Taxonomic Approach Points towards a Single-Species Hypothesis for Santolina (Asteraceae) in Corsica and Sardinia
Source: Biology (Basel). 2022 Feb 23;11(3):356. doi: 10.3390/biology11030356 (PMC8945001; doi:10.3390/biology11030356)
Supplement: Supplementary file 1 [file biology-11-00356-s001.zip › biology-1524779. Supplementary.pdf]

biology

MDPI

Table S1. GenBank accession numbers of the studied markers for each *Santolina* population. Voucher information is also provided.

Species	Population	Voucher	*ITS1-5,8S-ITS2*	*trnQ-rps16*	*trnH-psbA*	*trnL-trnF*	*trnS-trnG*	*psbM-trnD*	*rps15-ycf1*
S. chamaecyparissus	Le Luc	PI 034974	OL774773	OL771916	OL771891	OL771866	OL771941	OL771816	OL771841
S. chamaecyparissus	Le Luc	PI 034970 (CHA-LUC-2)	-	OL771917	OL771892	OL771867	OL771942	OL771817	OL771842
S. chamaecyparissus	Le Luc	PI 034970 (CHA-LUC-3)	-	OL771918	OL771893	OL771868	OL771943	OL771818	OL771843
S. corsica	Mont Pigno	PI 036636 (COR-LC-1)	OL774775	OL771919	OL771894	OL771869	OL771944	OL771819	OL771844
S. corsica	Mont Pigno	PI 036636 (COR-LC-2)	-	OL771920	OL771895	OL771870	OL771945	OL771820	OL771845
S. corsica	Mont Pigno	PI 036637 (COR-LC-3)	-	OL771921	OL771896	OL771871	OL771946	OL771821	OL771846
S. corsica	Monte Albo	PI 036122	OL774774	OL771922	OL771897	OL771872	OL771947	OL771822	OL771847
S. corsica	Monte Albo	PI 036123	-	OL771923	OL771898	OL771873	OL771948	OL771823	OL771848
S. corsica	Monte Albo	PI 036124 (COR-ALB-3)	-	OL771924	OL771899	OL771874	OL771949	OL771824	OL771849
S. insularis	San Benedetto	PI 036082	OL774779	OL771925	OL771900	OL771875	OL771950	OL771825	OL771850
S. insularis	San Benedetto	PI 036080 (INS-LC-2)	-	OL771926	OL771901	OL771876	OL771951	OL771826	OL771851
S. insularis	San Benedetto	PI 036080 (INS-LC-3)	-	OL771927	OL771902	OL771877	OL771952	OL771827	OL771852
S. insularis	Buggerru	PI 036613 (INS-BUG-1)	OL774776	OL771928	OL771903	OL771878	OL771953	OL771828	OL771853
S. insularis	Buggerru	PI 036613 (INS-BUG-2)	-	OL771929	OL771904	OL771879	OL771954	OL771829	OL771854
S. insularis	Buggerru	PI 036614	-	OL771930	OL771905	OL771880	OL771955	OL771830	OL771855
S. insularis	Laconi	PI 036057	OL774778	OL771931	OL771906	OL771881	OL771956	OL771831	OL771856
S. insularis	Laconi	PI 036058	-	OL771932	OL771907	OL771882	OL771957	OL771832	OL771857
S. insularis	Laconi	PI 036060	-	OL771933	OL771908	OL771883	OL771958	OL771833	OL771858
S. insularis	Monte Spada	PI 036106	OL774777	OL771934	OL771909	OL771884	OL771959	OL771834	OL771859
S. insularis	Monte Spada	PI 036107	-	OL771935	OL771910	OL771885	OL771960	OL771835	OL771860
S. insularis	Monte Spada	PI 036108	-	OL771936	OL771911	OL771886	OL771961	OL771836	OL771861
S. insularis	Monte Corrasi	PI 036663 (INS-OLI-1)	OL774780	OL771937	OL771912	OL771887	OL771962	OL771837	OL771862
S. insularis	Monte Corrasi	PI 036663 (INS-OLI-2)	-	OL771938	OL771913	OL771888	OL771963	OL771838	OL771863
S. insularis	Monte Corrasi	PI 036662 (INS-OLI-3)	-	OL771939	OL771914	OL771889	OL771964	OL771839	OL771864

Table S2. Variables used by the LDA in the cypsela morpho-colorimetric analysis. 'Tolerance' and 'F-to remove' values are reported. 'Feret' is the largest axis length.

Code	Description of the character	Tolerance	F-to remove
Area	Area defined by the perimeter	0.003	16.733
ArEquivD	Area equivalent diameter = $\sqrt{[(4/\pi) \times \text{Area}]}$	0.002	14.795
Rectang	Rectangularity = Area/ArBBox	0.083	11.314
Solidity	Solidity = Area/Area of the convex hull	0.113	8.943
MBCRadius	Length of the radius of the minimum bounding circle	0.002	8.248
ArBBox	Area of the bounding box along the Feret diameter	0.006	7.799
AspRatio	Feret/Breadth	0.029	7.712
CHull	Convex hull or convex polygon calculated from pixel centres	0.002	7.055
ModRatio	(2 × MinR)/Feret	0.005	6.321
Sphericity	Sphericity = MinR/MaxR	0.006	5.291
Circ	Circularity = $4 \times \pi \times \text{Area}/\text{Perimeter}^2$	0.168	4.851
MaxR	Radius of the enclosing circle centred at the middle of mass	0.005	4.642
MinR	Radius of the inscribed circle centred at the middle of mass	0.012	4.386
CArea	Area of the convex hull polygon	0.002	4.182

Table S3. Morphological characters that are significantly different among *Santolina* populations. Capital letters represent homoscedastic variables (Bartlett test with $p > 0.05$). Lower case letters represent non homoscedastic variables. Here follows the list of letters and related variables. In brackets, the times each variable occurs in the table is reported. fs_length = A (3), br_ratio = b (12), dist_cap_lf = c (6), fs_node_length = a (1), ss_length = D (5), ss_node_length = d (1), cap_diam = e (8), sq_ext_length = F (2), sq_int_length = G (9), sq_int_width = h (6), sq_if_length = i (8), flower_length = L (10), ssl_length = m (11), ssl_petiole_length = o (2), ssl_seg_length = f (1), ssl_seg_width = p (7), ssl_seg_dist = Q (2), fsl_length = r (7), fsl_petiole_length = S (10), fsl_seg_width = t (3). Character acronyms as in Table 4.

**Populations**	***S. corsica* Monte Albo**	***S. corsica* Mont Pigno**	***S. insularis* San Benedetto**	***S. insularis* Buggerru**	***S. insularis* Monte Spada**	***S. insularis* Monte Corrasi**	***S. insularis* Laconi**
***S. corsica* Mont Pigno**	b/e/h/p/q/L/S/v/w/Ò						
***S. insularis* San Benedetto**	b/c/v/x	A/D/e/F/G/h/L/p/r/S/t/w					
***S. insularis* Buggerru**	b/i/m/S/U/v/x/Ò	G/f/p/q/Q/t/U	A/a/c/D/e/i/m/S/Ò				
***S. insularis* Monte Spada**	G/m/o/U/j	b/e/F/G/L/m/p/U/v/j/x/w/Ò	b/G/m/o/r/S/v/j/x/w	b/c/D/G/i/j/x/w/Ò			
***S. insularis* Monte Corrasi**	m/z/k/S	b/e/h/L/p/r/v/z/j/x/Ò	A/b/D/i/m/r/S/v/z/k/j/x	b/c/i/Q/v/z/j/x/Ò	G/w		
***S. insularis* Laconi**	L/m/U/j	b/D/e/p/j/U/x/y/w/Ò	m/r/S/j/x/w	A/b/c/d/D/j/x/Ò/w	b/G	D/i/L/z/y/w	
***S. chamaecyparissus* Le Luc**	G/L/m/j/x	b/c/e/G/h/L/p/r/S/t/j/x/w/Ò	L/m/r/j/x	G/c/e/h/i/L/S/j/x/Ò	L/S/j/x	G/i/h/L/S/j/x	G/L/S/j/x/y

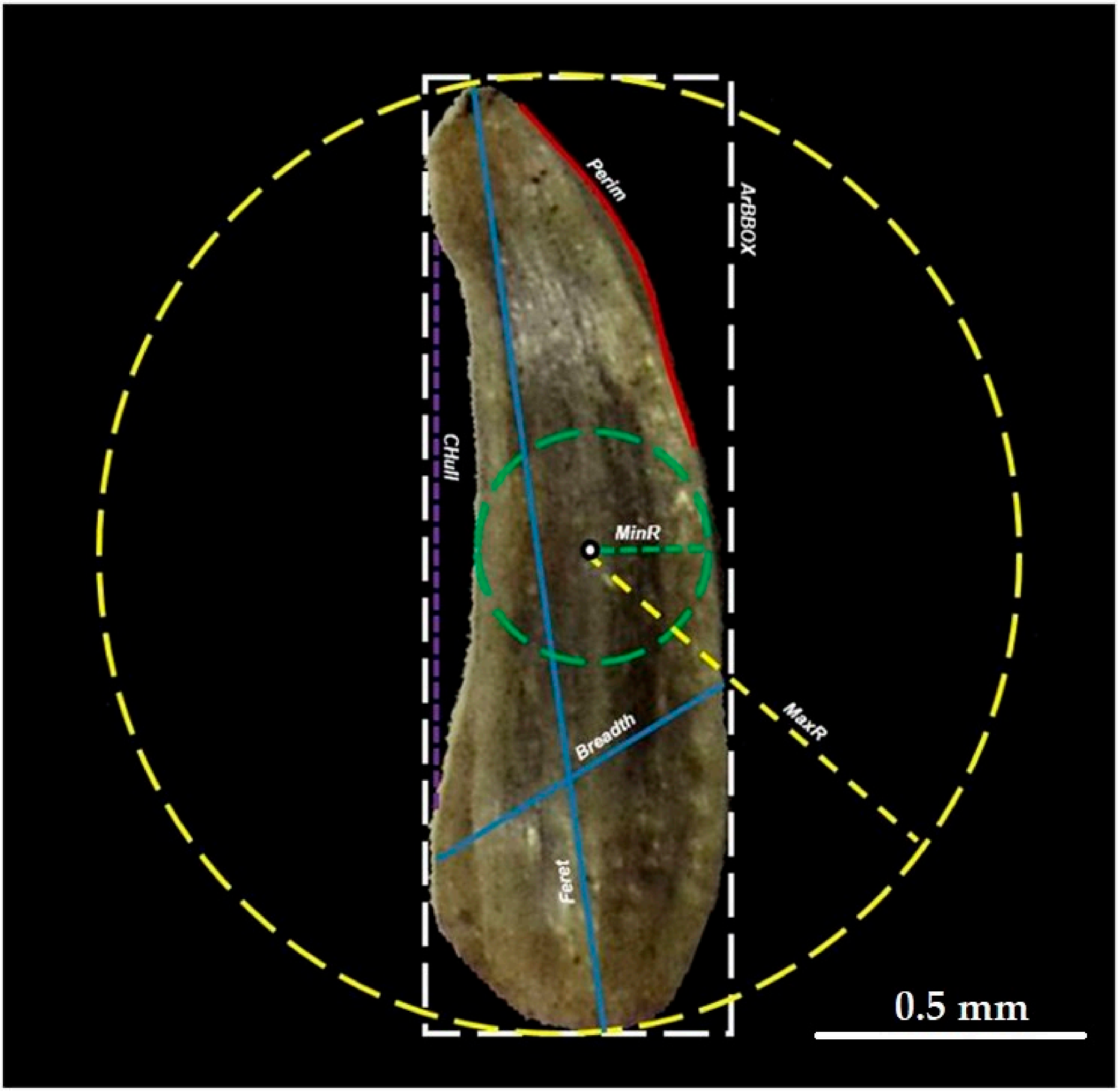

Figure S1. Graphic explanatory representation of some morphometric variables used in the cypsela morpho-colorimetric approach.

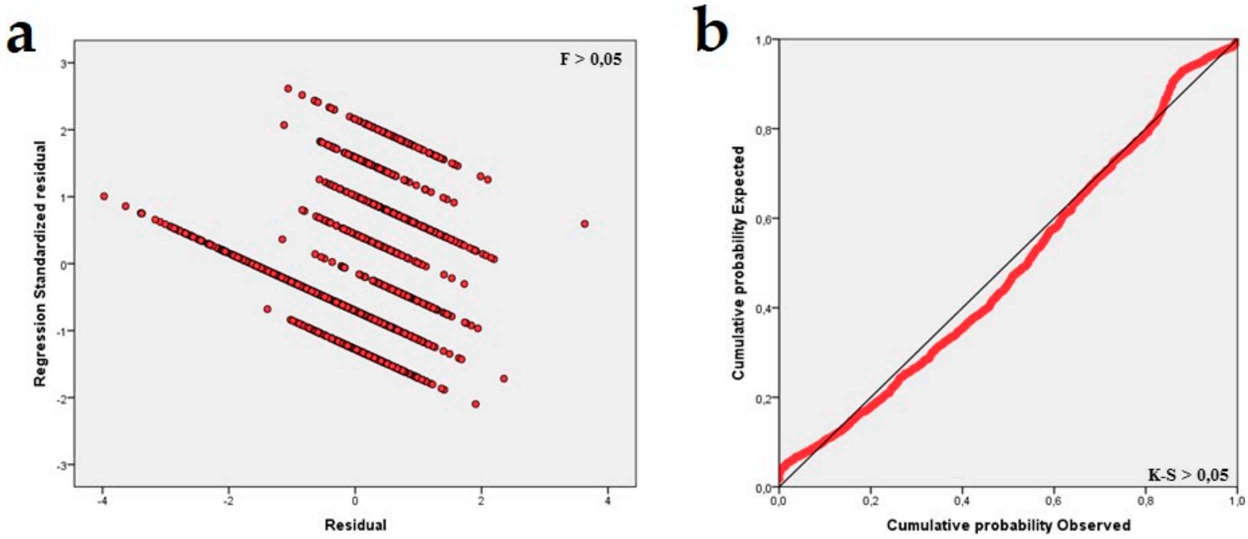

Figure S2. Cypsela morpho-colorimetric analysis. Dispersion plot of the standardized residuals tested with Levene's test (F) (a); normal probability plot (P-P) tested with Kolmogorov–Smirnov's test (K–S) (b).
